# Longitudinal metabolic imaging of hepatocellular carcinoma in transgenic mouse models identifies acylcarnitine as a potential biomarker for early detection

**DOI:** 10.1038/srep20299

**Published:** 2016-02-02

**Authors:** Jadegoud Yaligar, Wei Wei. Teoh, Rashidah Othman, Sanjay Kumar Verma, Beng Hooi Phang, Swee Shean Lee, Who Whong Wang, Han Chong Toh, Venkatesh Gopalan, Kanaga Sabapathy, S. Sendhil Velan

**Affiliations:** 1 Laboratory of Molecular Imaging, Singapore Bioimaging Consortium, Agency for Science, Technology and Research, Singapore; 2 Laboratory of Molecular Carcinogenesis, National Cancer Center, Singapore; 3 Laboratory of Cell Therapy and cancer Vaccine, National Cancer Center, Singapore

## Abstract

The cumulative effects of hepatic injury due to hepatitis B virus (HBV) infections and aflatoxin-B_1_ (AFB_1_) exposure are the major risk factors of HCC. Understanding early metabolic changes involving these risk factors in an animal model closely resembling human hepatocellular carcinoma (HCC) is critical for biomarker discovery and disease therapeutics. We have used the hepatitis B surface antigen (HBsAg) transgenic mouse model that mimics HBV carriers with and without AFB1 treatment. We investigated early metabolic changes from preneoplastic state to HCC by non-invasive longitudinal imaging in three HCC groups of mice: HBsAg + AFB_1_(Gp-I), AFB_1_ alone (Gp-II), HBsAg alone (Gp-III) and a control group (wild-type untreated; Gp-IV). For the first time, we have identified acylcarnitine signals *in vivo* in the liver prior to the histological manifestation of the tumors in all three groups. Acylcarnitine concentration increased with increase in tumor growth in all HCC mouse models, indicating elevated metabolic activity and increased cell turnover. This was confirmed in a pilot study using human serum from HCC patients, which revealed a higher concentration of acylcarnitine compared with normal subjects. Translational clinical studies can be designed to detect acylcarnitine in patients with high risk factors for HCC.

Hepatocellular carcinoma (HCC) is a devastating form of cancer worldwide, with a poor survival rate^1^. The risk factors associated with HCC include hepatitis B (HBV) and hepatitis C (HCV) viral infections, and aflatoxin-B_1_ (AFB_1_) exposure^2^. Epidemiological studies have suggested a strong correlation between AFB_1_ exposure and a signature mutation on the tumor suppressor gene *p*53^3^,^4^,^5^. The combined effect of HBV infection and AFB_1_ exposure involving *p*53 mutations increases the risk of HCC in humans. Earlier *in vivo* and *in vitro* studies on metabolic investigations of HCC utilized chemically induced or subcutaneous tumors in rodent models^6^,^7^,^8^,^9^,^10^. However, the *p*53 mutations have not been incorporated in these spontaneous HCC models, which lack in mimicking the appropriate human environment. Although non-invasive imaging modalities, such as ultrasound (US), computed tomography (CT) and magnetic resonance imaging (MRI) techniques, have improved the detection and localization of HCC at later stages, they are not specific in early detection of HCC. Hence understanding metabolic changes at early stage of HCC could facilitate development of biomarkers. Noninvasive MRI and magnetic resonance spectroscopic (MRS) approaches permits longitudinal imaging with wide range of techniques for identification of biomarkers that can be translated to humans^11^. Abnormal choline is a metabolic hallmark associated with oncogenesis and tumor progression^12^. An elevated level of choline and phosphocholine in malignant lesions has been established as a diagnostic marker for cancer^13^,^14^. Acylcarnitine plays an essential role by enabling long chain fatty acids to enter the mitochondria matrix for β-oxidation^15^ and abnormal levels have been associated with septic patients^16^ and endocrine disorders^17^. Lipids are vital for energy homeostasis, organ physiology and other aspects of cellular biology, and are linked to pathological processes, such as inflammation, obesity and liver disease^18^. Hepatic cellular damage may cause impairment in liver function, and can lead to alterations in lipid metabolism and subsequently plays a vital role in the development of HCC. Abnormal metabolic changes in tumor tissues are also accompanied by changes in cellularity, and the mobility of water molecules is altered due to their interactions with cell membranes and macromolecules. Diffusion tensor imaging (DTI) enables qualitative and quantitative characterization of tissue microstructural changes^19^,^20^. In highly cellular tumor tissues, there will be changes in the volume ratio or physical nature of intra- and extracellular spaces that restrict the water diffusion^20^.

Some of the commonly used screening procedures for patients at risk include serum α-fetoprotein (AFP) levels. Its specificity is very limited because many other liver diseases can also result in elevated levels of AFP^21^. The imaging technologies for diagnosis of liver cancer, such as MRI, CT and US, permits detection of tumors >1 mm. MR image confirmation of a neoplasm usually occurs at later stages of tumor growth. In some cases, neoplasms that are beyond the imaging sensitivity limits require a confirmation by invasive biopsy techniques. Therefore, sensitive and specific biomarkers for the early (preneoplastic stage) detection of HCC might help in prevention and better management of HCC. In this study, we used four groups of mice (three HCC groups and one control group) models which closely resemble human HCC in their histopathological features and upstream regulators^22^. We investigated the early metabolic changes from normal liver to malignant stage involving independent and cumulative risk factors of hepatitis B, and AFB_1_ exposure. Longitudinal MRI and MRS were performed to characterize the HCC and studied the changes in lipid composition, and their correlation to tumor malignancy.

## Results

### Tumor growth kinetics

**Figure 1:**
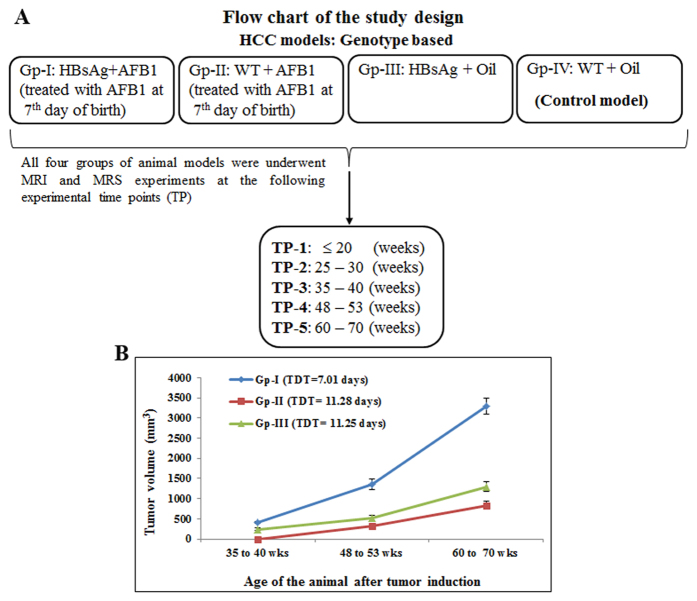
Flow chart of the study design and tumor growth kinetics of HCC and control Groups. (**A**) The flow chart for the study showing HCC and control mouse models. Gp-I HCC: HBsAg transgene + AFB_1_; Gp-II model: WT + AFB_1_; Gp-III model HBsAg transgene + Oil and Gp-IV control model: WT + oil. (**B**) Tumor growth kinetics of Gp-I, Gp-II, and Gp-III HCC models. The Gp-I, Gp-II animals did not show tumors until 30 weeks and Gp-III animals did not show tumors until 40 weeks of their age. The TDT (7.01 ± 0.38 weeks) of Gp-I tumors was significantly (*P* < 0.05) shorter compared to TDT of Gp-II and Gp-III tumors.

The flow chart of the study design is shown in Fig. 1A. In the current study, we have three groups of HCC models (Gp-I, II and III) and a control group (Gp-IV). Tumor volumes of the three HCC models (Gp-I, II and III) were measured to assess the tumor aggressiveness with age. Tumor growth kinetics of Gp-I–III at different weeks is shown in Fig. 1B. The Gp-IV animals (control group) did not develop tumors. Gp-I and III animals exhibited tumors at 35 weeks. The Gp-II tumors did not show tumors until 40 weeks. The tumor doubling time (TDT) for Gp-I, II, and III animals were 7.01 ± 0.38, 11.28 ± 0.47, and 11.25 ± 0.31 weeks, respectively. The TDT of Gp-I tumors was significantly shorter (*P* < 0.05) compared with the TDT of Gp-II and III tumors. The cumulative effect of risk factors HBsAg and AFB_1_ contributed to highly aggressive and proliferative tumor growth in Gp-I HCC compared with Gp-II and III HCC models.

### Metabolic characterization of HCC models

**Figure 2:**
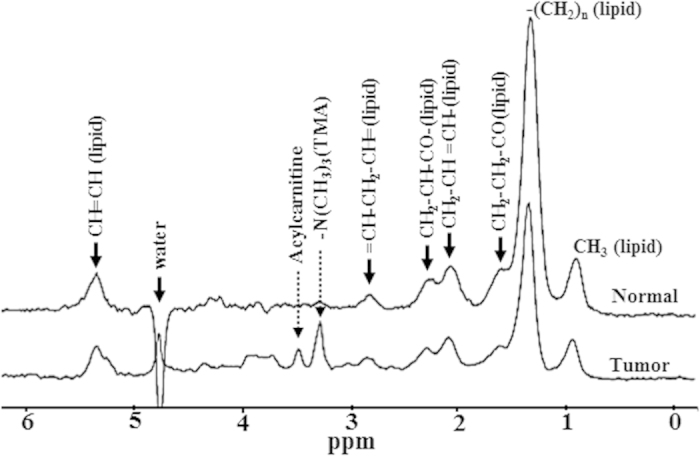
*In vivo*^1^H MRS of HCC and control groups. Localized *in vivo* spectra from Gp-I HCC tumor and Gp-IV normal liver. Dominant lipid resonances were assigned in the region of 0.8–5.3 ppm. The acylcarnitine and TMA resonances (choline) are highlighted in the tumor spectra.

**Figure 3:**
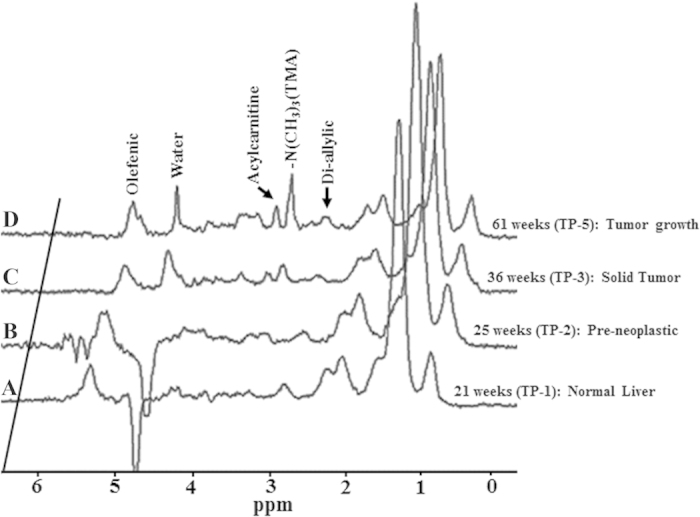
*In vivo* longitudinal spectra of Gp-I HCC model at different stages of HCC growth and normal liver. (A) At 21weeks of age no tumors were formed in the liver and acylcarnitine was absent. (**B**) At 25 weeks acylcarnitine peak is seen prior to the histological manifestation of the tumor. (**C,D**) At 36 and 61 weeks, with increase in tumor growth acylcarnitine and choline concentrations were significantly increased.

**Figure 4:**
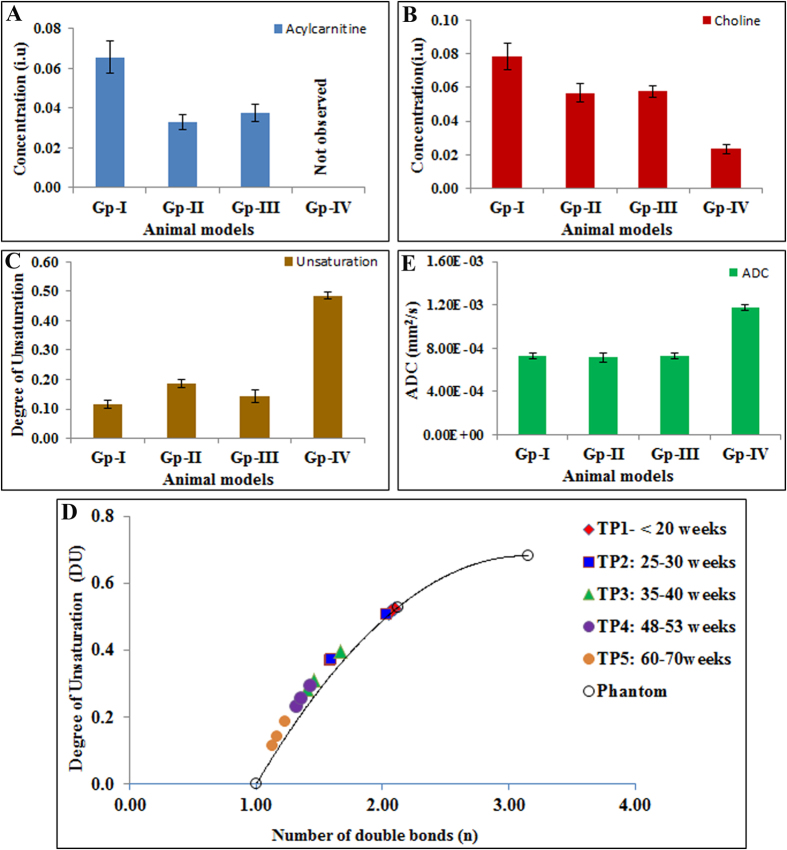
Concentrations of the acylcarnitine, choline, DU and ADCs from HCCs and control livers at 60–70 weeks. (**A**) Acylcarnitine levels in HCC models (Gp-I, II, III) and control group (Gp-IV). (**B**) Choline levels, (**C**) DU and (**E**) average ADCs for HCC and control group. (**D**) Correlation between DU and the number of double bonds in phantom and HCCs at different stages of tumor growth. At <20 weeks, the DU of HCC groups and control group were similar and comparable to the DU of linoleic acid (double bonds = 2). With increase in tumor growth the DU significantly decreased corresponding to the number of double bonds = 1 (oleic acid).

Figure 2 shows representative *in vivo* proton spectra obtained from the HCC tumor of Gp-I and control livers of Gp-IV animals. Prominent resonances from lipids and trimethylamine (TMA) protons of choline are indicated in the spectra. We also observed the acylcarnitine signal from γ CH_2_ at 3.51 ppm in HCC of Gp-I, II, and III animals, where as acylcarnitine was not observed in the livers of control group (Gp-IV). The presence of acylcarnitine in tumors was further confirmed by performing 2D COSY high resolution magic angle spinning spectroscopy (HRMAS) of intact tumor tissues (supplementary Fig. 1). The *J* coupling mediated cross peaks between β CH group with α, γ CH_2_ groups of acylcarnitine are highlighted. Figure 3A–D, show the longitudinal liver spectra from the same animal from Gp-I HCC at different stages (21–61 weeks) of tumor growth. Figure 3A shows the normal liver spectrum from 21 week old mouse and the concentration of choline was very minimal and acylcarnitine was not observed. At 25 weeks (Fig. 3B) of age, although there were no visible tumors found in the liver, acylcarnitine peak was identified in the liver (preneoplastic tissue). At 36 weeks (Fig. 3C), the concentrations of acylcarnitine and choline in HCC tumors (Gp-I and III) were significantly (*P* < 0.05) higher compared with the preneoplastic state at 25 weeks. With an increase in age, there was a significant increase in tumor volume. At 61 weeks (Fig. 3D) the acylcarnitine and choline concentrations in all HCC groups were significantly (*P* < 0.05) higher compared with their previous time points. Figure 4A,B show the concentrations of acylcarnitine and choline for Gp-I, GpII and Gp III HCC at 60–70 weeks. Longitudinal assessment of acylcarnitine and choline for all groups (Gp-I–IV) during 20–70 weeks is shown in Table 1. The concentration of acylcarnitine and choline in Gp-I tumors was significantly higher (*P* < 0.05) than both Gp-II and Gp-III tumors. In Gp-IV animals (control model), acylcarnitine was absent at all time points (20–70 weeks), and choline concentrations were significantly lower (*P* < 0.05) than the choline content of HCC models (Table 1).

### Degree of unsaturation in HCC models

Figure 4C shows the average degree of unsaturation (DU) from all four groups (Gp-I–IV) at 60–70 weeks. The average DU of Gp-I, II and III HCC tumors were significantly lower (*P* < 0.005) than the normal liver. The DU for all three HCC groups and the control group at different time points (20–70 weeks) are shown in Table 2. The DU of the tumors was decreased significantly with increase in tumor volume. Figure 4D shows the correlation between DU from HCCs at different stages of tumor growth and the number of double bonds in phantom. DU of all three HCC groups and control liver did not differ significantly (*P* > 0.05) before 20 weeks. At 25–30 weeks, although the tumors were not observed, the DU of HCCs (Gp-I and III) were significantly reduced (*P* < 0.05) compared with normal liver (Table 2). With increase in tumor volume from 35–70 weeks the DU of HCCs were significantly reduced and the DU values correlated to the presence of one double bond based on our phantom measurements.

### Diffusion weighted imaging of HCC

Tumor apparent diffusion coefficient (ADC) values for all HCC groups at different stages are shown in Table 2. The average ADC (Fig. 4E) values measured in tumor areas from HCC (Gp-I, II and III) were significantly lower (*P* < 0.005) than the control groups (Gp-IV) at 60–70 weeks. Prior to the formation of tumors (~20 weeks) in the HCC groups, the ADC values were similar to the normal liver. With increase in tumor volume the ADC values of all HCC groups at different stages of tumor growth were significantly lower (*P* < 0.005) than the normal liver.

### Histology and mRNA analysis of HCC models

Histology analysis of the tumors has been described in our earlier work^22^. Gp-I HCC showed more aggressive histological features corresponding to severe carcinoma compared with Gp-II and Gp-III. HCC is represented either as a trabecular or solid form compressing adjacent normal parenchyma, with pleomorphic nuclei and aberrant mitotic figures. The Gp-III tumor tissues showed large hepatocytes of varying size and with a shape of pleomorphic nuclear and cell foci. The Gp-IV liver tissues had normal liver characteristics without undergoing any size and shape transformation. The real-time PCR mRNA expression analysis stearoyl-CoA desaturase 1 (*SCD1)* gene was performed using RNA isolated from HCC groups (Gp-I–III) and control liver. The *SCD1* was significantly (*P* < 0.05) up-regulated in tumors of Gp-I, Gp-II and Gp-III by 2.91, 2.20 and 2.43 fold compared to the control liver.

### Serum analysis of HCC patients

**Figure 5:**
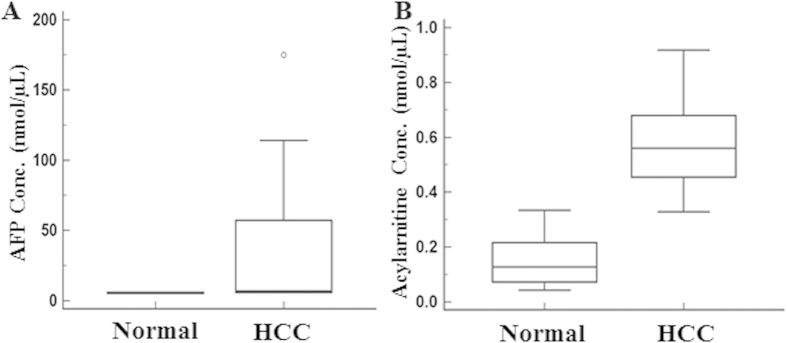
Serum acylcarnitine and AFP in HCC patients and normal subjects. (**A**) AFP and (**B**) Acylcarnitine concentrations in serum from HCC patients and normal subjects. Serum acylcarnitine in HCC patients were significantly higher (*P* < 0.005) than normal subjects.

To evaluate if acetylcarnitine can be detected in human HCC patients, in a pilot study we analyzed the acylcarnitine concentration in serum of HCC patients and normal subjects (Fig. 5). The acylcarnitine levels were significantly higher (*P* < 0.005) in HCC patients (0.57 nmol/μL) compared to normal subjects (0.12 nmol/μL). Importantly, all the patients showed elevated carnitine, whereas the AFP was elevated in only 40% of patients, highlighting the potential utility of carnitine as a biomarker for HCC.

## Discussion

The cumulative effects of hepatic injury due to HBV infection and AFB_1_ exposure are the major risk factors of HCC in humans^2^ and mice^23^. Understanding the independent risk factors and early metabolic changes involving HBV, and AFB_1_ is essential for biomarker discovery and disease therapeutics. We studied the metabolic changes in HCC associated with cumulative and independent risk factors of HBsAg and AFB_1_ exposure. In this study Gp-I HCC mice represent the combined risk factors involving HBsAg and AFB_1_ exposure. The aggressive nature of tumors in Gp-I is evident from the tumor growth kinetics (Fig. 1B) compared with Gp-II and III HCC mice. The tumor doubling time of Gp-I HCC mice was significantly (*P* < 0.05) shorter compared with Gp-II and III. Unlike Gp-II and III animals, the Gp-I mouse livers were completely transformed into HCC by 60–70 weeks. This implies the synergistic effects of HBsAg and AFB_1_ in the formation of aggressive HCC. Aggressive tumors are metabolically active and show altered metabolite composition compared with normal liver. In the current study, we observed the acylcarnitine in all three groups of tumor mice in addition to choline. At the age of 25–30 weeks acylcarnitine was observed at the initiation stage, prior to the histological manifestation (preneoplastic stage) of the tumors in Gp-I and III HCC mice. Formation of acylcarnitine is essential for energy metabolism for facilitating the transport of long chain fatty acids (as acyl-coenzyme A) to enter the mitochondria for β-oxidation to produce adenosine triphosphate^24^,^25^. Carnitine metabolism can be impaired in multiple ways in animals or humans under chronic liver conditions (HCC and cirrhosis)^25^,^26^. Carnitine palmitoyltransferase 1 (*CPT 1*) is responsible for the formation of fatty-acylcarnitine by catalyzing the transfer of the acyl group from long-chain fatty acyl coenzyme A (CoA) to L-carnitine^27^,^28^,^29^. Accumulation of fatty-acylcarnitines in preneoplastic tissues of Gp-I and III mice (at 25–30 weeks) and in Gp-II animals (at 35–40 weeks) is due to the upregulation of *CPT 1* and may lead to the increased availability of the fatty acids to meet the energy demands of the preneoplastic tissue which was predisposed to tumor formation. The acylcarnitine concentrations increased with the increase in tumor volume in all three HCC groups. This reflects the elevated metabolic activity and increased cell turnover of the tumor cells by producing more acylcarnitine with upregulation of *CPT 1* to fuel the mitochondrial activity for maintaining the rapid tumor growth. This situation is complimented by down regulation of carnitine palmitoyltransferase 2 (*CPT 2*), which has impaired catalyzation of fatty-acylcarnitine to fatty acylCoA and free carnitine. *CPT 2* is actually responsible for catalyzing fatty-acylcarnitine to fatty acylCoA, which were involved in the mitochondrial β-oxidation, thereby releasing carnitine back into the cytoplasm. The accumulation of acylcarnitine in HCCs with increased tumor volume might be due to the cumulative effect of upregulation of *CPT 1* and down regulation of *CPT 2* genes. Zhou *et al.*^30^ have reported the increase of long chain acylcarnitines with deficiency of *CPT 2* in plasma of HCC patients. In the current study, we observed 3.5 fold increase in acylcarnitine and choline in Gp-I HCC mice compared with its preneoplastic tissue, whereas, in Gp-II and III HCCs, the acylcarnitine and choline increased by 2 fold. This aggressive metabolic state of Gp-I HCC compared with Gp-II and III HCC mice can be explained by the combined synergistic effects of HBsAg and AFB_1_ risk factors. In a pilot study we further evaluated the concentration of acylcarnitine in serum obtained from HCC patients and normal subjects. As shown in Fig. 5 the acylcarnitine was significantly higher in HCC patients compared to the normal subjects and also more specific compared to traditional measurement of AFP. Large scale patient studies will be required to determine the potential utility of acylcarnitine as a biomarker of HCC in humans. Earlier study on Nigerian population also reported significantly higher levels of acylcarnitine in urine samples of HCC patients^31^.

The reprogramming of lipid metabolism has been established as an important characteristic of neoplasms^32^. Fatty acids serve as signaling molecules involved in cell proliferation^33^. Alterations in fatty acid profiles triggered by cell proliferation result in a wide range of effects influencing the integrity of the cellular membranes, and also effects membrane structure, fluidity and the affinity of growth factor receptors. Major metabolic alterations involving lipid metabolism and changes in lipid composition associated with HCC have been reported^34^. In the current study, we have used the DU as a measure of change in lipid composition with increased tumor growth. Before tumor formation (around 20 weeks), the DU of HCC groups (Gp-I–III) were similar to the control group (Gp-IV) and matched to DU of linoleic acid with two double bonds (Fig. 4D) indicating the abundant availability of linoleic acid before tumor formation. At 25–30 weeks, even before the formation of neoplasms we observed significant alterations in lipid composition as evidenced by the significant decrease in the DUs of HCC mice. During this preneoplastic stage, the tissue was more prone to undergo chain of crucial metabolic changes attributed to aggressive progression of the neoplasm. Sandor *et al.*^35^ have reported the alterations in fatty acid metabolism resulting in increase of acylcarnitines. In the present study, the alternations in lipid composition were in concurrent with elevated acylcarnitine in the preneoplastic tissues of all three HCC groups. As shown in Table 2 with the progression of tumor growth from 30–70 weeks, the DU of the Gp-I, II, and III HCCs was abruptly reduced, indicating reduced availability of polyunsaturated lipids. This alteration in fatty acid composition during tumor growth might be due to changes in desaturase expression or activities, and the extent of lipid alterations. The change in lipid composition with increased mono-unsaturated lipids (oleic acid) as the normal liver transforms into HCC was supported by the upregulation of *SCD1* levels in HCC. The desaturase enzymes, stearoyl-CoA desaturase (SCD) involved in fatty acid synthesis was known to contribute high oleic acid levels^36^. The abundance of mono-unsaturated lipid (oleic acid) in tumors was supported by correlating the DU with the number of double bonds as a function of tumor growth (Fig. 4D). The decrease in DU with increased tumor growth corresponded to the number of double bonds of n = 1 and indicates the increased abundance of oleic acid. Proliferating cells that lack oleic acid fail to grow because they cannot initiate chromosomal DNA replication. An increase in oleic acid content in tumors is vital for cell proliferation^37^,^38^. Our observations were in agreement with the study conducted by Griffiths *et al.*^39^ in a TGFα/c-myc mouse HCC model. They have reported a significant decrease in DU in this model and also showed a significant increase in oleic acid along with major alterations in unsaturated fatty acids during hepatocarcinogenesis. Another study reported an increased total lipid profile with a decrease of lipid polyunsaturation in liver biopsies with chronic hepatitis C^40^.

Change in cellularity of the HCCs is evidenced by significantly reduced ADC in Gp-I, II and III. Under pathological conditions, the rigidity of the extracellular space and high density of hydrophobic cellular membranes in HCC tissues (Gp-I–III) restrict the apparent diffusion of water, thereby resulting in lower ADCs.

In Summary, we have identified the accumulation of acylcarnitine in preneoplastic stages of HCC as a characteristic feature of early metabolic changes in HCC. Acylcarnitine could be considered as model specific HCC biomarker because of multiple risk factors involved in forming HCC. To the best of our knowledge, this is the first study showing an early metabolite marker acylcarnitine, prior to the histological manifestation of the tumors. This emphasizes that acylcarnitine might be a potential biomarker that can be detected *in vivo* in HCC, though a larger cohort study would be required to establish the exact range to determine positivity. Pre-clinical studies involving drug therapeutic interventions can be further explored by monitoring acylcarnitine levels in growing tumors and further clinical studies can be explored on selected high risk subjects as a translational step in establishing this marker in human patients.

## Methods

### Animal models of HCC

All the animal studies were carried out in accordance with approved guidelines and all experimental protocols were approved by the Institutional Animal Care and Use Committee (IACUC) of Sing Health and the Biological Resource Center, Agency for science, technology and research (A*STAR), Singapore. HBsAg transgenic mice, C57BL/6 J-Tg (Alb1HBV) 44Bri/J (Jackson laboratory, USA) and their littermate C57BL/6 J wild-type (WT) controls were given a single intraperitoneal injection of AFB_1_ (6 μg/g body weight [19 nmol/g]) in trioctanoin-containing 6% DMSO or corn oil on day 7 (D7) after birth, as previously described^22^,^41^. In this study, we used three groups of HCC models and one control group. Group I (Gp-I) involves HBsAg + AFB_1_ (n = 10) and is obtained by treating HBsAg (Hepatitis B surface Antigen as a transgene) expressed in transgenic mouse with AFB_1_. Group II (Gp-II) involves WT + AFB_1_ (n = 6) obtained by treating WT type mice with AFB_1_. Group III (Gp-III) involves HBsAg + Oil (n = 6) (HBsAg only) obtained by treating HBsAg mice with oil. Group IV (Gp-IV) involves WT + Oil (n = 8) and is obtained by treating WT type mice with oil. Longitudinal *in vivo* measurements were performed on all four groups (Gp-I, II, III, and IV) at different experimental time points (TP-1: ≤20 weeks, TP-2: 25–30 weeks, TP-3: 35–40 weeks, TP-4: 48–53 weeks and TP-5: 60–70 weeks). Liver tissues including nodules and uninflamed normal areas were subjected to histology after terminal *in vivo* experiments.

### *In vivo* MRI and spectroscopy

All animals were subjected to MRI and localized MRS. Prior to *in vivo* experiments; animals were initially anesthetized with 2.5% isofluorane in a dedicated chamber. During the course of MRI/MRS experiments, isofluorane levels were reduced to 1.5–2.0% in a combination of medical air and medical oxygen. *In vivo* imaging and spectroscopic experiments were performed using a 7 T MRI/MRS scanner (ClinScan, Bruker BioSpin GmbH, Ettlingen, Germany) with Siemens VB15 platform. For high resolution diffusion tensor imaging, a 35-mm volume transmit/receive mouse body coil was used, and for spectroscopic applications 20 mm surface receive only coil was used in combination with volume transmit body coil. To localize the tumor in the liver, T_1_ and T_2_ weighted MRI were performed. The T_1_ weighted images were acquired by using gradient echo sequence with TR = 500 ms, TE = 3.2 ms, matrix size = 256 × 256, slice thickness = 1 mm, and averages = 3. The T_2_ weighted images were acquired using turbo spin echo (TSE) sequence with TR = 2956 ms, TE = 29 ms, and number of averages = 2. Diffusion weighted images were acquired on the entire liver using spin echo-based echo-planar imaging (SE-EPI) with TR = 2800 ms, TE = 30 ms, FOV = 35 × 35 mm, directions = 20, b values = 0, 1000 s/mm^2^, averages = 4, matrix size = 256 × 256, and slice thickness = 1 mm. A Java-based ImageJ plugin was utilized for processing the DTI and ROI analyses^42^,^43^,^44^. Respiration and body temperature was monitored using a physiological monitoring system (ML880 16/30 power lab system, AD Instruments, Spechbach, Germany). The temperature probe was placed in the rectum of the mice, and body temperature was maintained at 37 °C by circulating hot water through a mouse cradle on which the animal was resting. Volume localized point resolved spectroscopy sequence (PRESS) experiments were performed on a 2 × 2 × 2 mm^3^ voxel within the liver using TR = 4000 ms, TE = 13 ms, 128 averages, and 2048 complex points with a spectral width of 3500 Hz. A respiratory triggered gating module was incorporated into the PRESS sequence with a trigger delay of 20 ms and an animal breathing was stabilized at 85–90 cycles/min. Water unsuppressed spectrum was acquired under identical conditions with similar parameters and 4 averages.

### Data analysis and statistics

High resolution T_2_ and T_1_ weighted images were used to compute the tumor volumes and tumor doubling time (TDT). Tumor volumes (mm^3^) were computed by using Medical Image Processing, Analysis and Visualization Tool (MIPAV - v7.0.1)^45^. The tumor doubling time (TDT) was calculated by taking into account the initial and final volume of the tumor and, the time interval between the two successive volumes^46^,^47^. Spectroscopic data were processed and analyzed using linear combination model fitting approach^48^. Metabolites concentrations for all the groups (Gp-1, II, III and IV) were estimated as ratio with unsuppressed water signal. The results are expressed as the mean ± SEM (standard error of the mean). The degree of unsaturation (DU) was estimated from the MRS data using the ratio of bi-allylic (CH_2_ peak at 2.7 ppm) signal to the olefinic (CH = CH peak at 5.3 ppm) signal^39^. Estimation of the DU was validated by developing the calibration curve using standard fatty acid samples, oleic acid (18:1), linoleic acid (18:2) and linolenic acid (18:3), which differ in the number of double bonds (unsaturation). The DU from the *in vivo* data (tumor and normal liver) was plotted in the calibration curve to evaluate the changes in lipid unsaturation during the tumor growth. The relationship between the DU and number of double bonds is, n = −1/ (DU − 1). Statistical analysis using non parametric Mann Whitney was performed using MedCalc® version 12.7.7.0. Differences were considered significant at *P < *0.05.

### mRNA analysis

Total RNA was extracted from the liver samples using trizol reagent (Invitrogen) and treated with DNase I prior to cDNA conversion using the revertAid H minus first strand cDNA synthesis kit (Fermentas, USA) with oligo d(T) 18 primer according to manufacturer’s instructions. For real time qPCR, cDNA samples were analyzed in triplicates using the SYBR® Green PCR Master Mix reagent kit (Applied Biosystems) on a StepOnePlus™ Real-Time PCR System (Applied Biosystems). Relative mRNA levels were calculated and normalized to glyceraldehyde 3-phosphate dehydrogenase GAPDH (CAAGGTCATCCATGACAACTTTG) and (GGCCATCCACAGTCTTCTGA), used as an endogenous control gene. The primer sequences used were as follows: stearoyl-CoA desaturase 1 SCD1; (CCTACGACAAGAACATTCAATCTC) and (TTGATGTGCCAGCGGTACTCACTG).

### Serum analysis of HCC patients

Human serum from HCC patients (n = 14) and normal subjects (n = 6) were obtained through Singhealth Institutional Ethics Committee approval. Acylcarnitine and alpha-fetoprotein (AFP) were analyzed by Abcam kit according to manufacturer’s instruction (Abcam, Ab83392).

## Additional Information

**How to cite this article**: Yaligar, J. *et al.* Longitudinal metabolic imaging of hepatocellular carcinoma in transgenic mouse models identifies acylcarnitine as a potential biomarker for early detection. *Sci. Rep.* **6**, 20299; doi: 10.1038/srep20299 (2016).

## Supplementary Material

Supplementary Information

## Figures and Tables

**Table 1:** Concentrations of acylcarnitine and choline from HCC (Gp-I–Gp-III) and control groups at different experimental time points (TP).

Animal groups	Experimental time points (TP)									
	≤20 weeks		25–30 weeks		35–40 weeks		48–50 weeks		60–70 weeks	
	(TP-1)		(TP-2)		(TP-3)		(TP-4)		(TP-5)	
	Carnitine	Choline	Carnitine	Choline	Carnitine	Choline	Carnitine	Choline	Carnitine	Choline
Gp-I	0	0.02 ± 0.002	0.02 ± 0.005	0.024 ± 0.004	0.033 ± 0.003	0.051 ± 0.002	0.035 ± 0.003	0.054 ± 0.003	0.066 ± 0.008	0.078 ± 0.008
Gp-II	0	0.018 ± 0.001	0	0.021 ± 0.003	0.018 ± 0.003	0.02 ± 0.003	0.026 ± 0.002	0.046 ± 0.001	0.033 ± 0.004	0.057 ± 0.006
Gp-III	0	0.02 ± 0.002	0.018 ± 0.001	0.022 ± 0.002	0.027 ± 0.002	0.046 ± 0.003	0.029 ± 0.003	0.051 ± 0.003	0.038 ± 0.004	0.058 ± 0.003
Gp-IV	0	0.016 ± 0.001	0	0.019 ± 0.001	0	0.021 ± 0.001	0	0.022 ± 0.002	0	0.023 ± 0.003

The acylcarnitine was significantly higher in tumors compared to the control group (*P* < 0.05). We observed significant (*P* < 0.05) increase in acylcarnitine between TP-2 and TP-3; TP-4 and TP-5 for all HCC groups (GP-I, GP-II and GP-III).

**Table 2:** Degree of unsaturation (DU) and apparent diffusion coefficient (ADC) from HCC (Gp-I–Gp-III) and control groups at different experimental time points (TP).

Animal groups	Experimental time points (TP)									
	≤20 weeks		25–30 weeks		35–40 weeks		48–50 weeks		60–70 weeks	
	(TP-1)		(TP-2)		(TP-3)		(TP-4)		(TP-5)	
	DU	ADC	DU	ADC	DU	ADC	DU	ADC	DU	ADC
Gp-I	0.524 ± 0.02	1.15 ± 0.06	0.369 ± 0.03	0.91 ± 0.05	0.285 ± 0.01	0.732 ± 0.02	0.237 ± 0.02	0.739 ± 0.03	0.117 ± 0.01	0.727 ± 0.03
Gp-II	0.512 ± 0.01	1.17 ± 0.04	0.506 ± 0.01	1.16 ± 0.05	0.399 ± 0.01	0.957 ± 0.01	0.297 ± 0.02	0.740 ± 0.04	0.186 ± 0.01	0.717 ± 0.04
Gp-III	0.518 ± 0.01	1.16 ± 0.02	0.373 ± 0.01	0.942 ± 0.01	0.313 ± 0.01	0.740 ± 0.04	0.259 ± 0.03	0.741 ± 0.04	0.144 ± 0.02	0.728 ± 0.03
Gp-IV	0.519 ± 0.01	1.15 ± 0.02	0.512 ± 0.01	1.20 ± 0.03	0.493 ± 0.01	1.16 ± 0.02	0.50 ± 0.01	1.18 ± 0.014	0.485 ± 0.01	1.17 ± 0.02

The DU of Gp-I, III tumors at TP-2–5 were significantly lower than the control group. The DU of Gp II tumors were significantly lower only at TP-3–5. The ADC (10^−3^  mm^2^ sec^−1^) of Gp-I, III tumors at TP- 3–5 were significantly lower compared to the control group, whereas the ADC of Gp II tumors was significantly lower only at TP 4, 5.
