# Supplementary material for: Longitudinal metabolic imaging of hepatocellular carcinoma in transgenic mouse models identifies acylcarnitine as a potential biomarker for early detection
Source: Sci Rep. 2016 Feb 2;6:20299. doi: 10.1038/srep20299 (PMC4735819; doi:10.1038/srep20299)
Supplement: Supplementary Information [file srep20299-s1.docx]

**Supplementary material**

**Longitudinal metabolic imaging of hepatocellular carcinoma in transgenic mouse models identifies altered acylcarnitine as a potential biomarker for early detection**

Jadegoud Yaligar, Wei Wei. Teoh, Rashidah Othman, Sanjay Kumar Verma, Beng Hooi Phang, Swee Shean Lee, Who Whong Wang, Han Chong Toh, Venkatesh Goplan, Kanaga Sabapathy, S. Sendhil Velan

**2D HRMAS COSY of tumor tissues**

Supplementary Fig. 1 shows the 2D HRMAS COSY spectrum obtained from the tumor tissue. NMR spectra were acquired in AVANCE III 400 spectrometer (Bruker, Germany) operating at ^1^H frequency of 400 MHz equipped with 4 mm double resonance HR MAS probe. Snap frozen tumor tissue (44 mg) was transferred to 4 mm ZrO_2_ rotor along with the 5 µl of D_2_O to provide a field-frequency lock. Sample preparation was performed under ice to minimize the changes in tissue metabolism after the biopsy. The HR MAS experiments were performed at 277 K and at a spin rate of 4 K Hz. One-dimensional ^1^H spectra were obtained using one pulse sequence with water suppression. Homonuclear (^1^H- ^1^H) two-dimensional COSY HR MAS spectra were acquired using sweep width of 6996 Hz in both dimensions, 256 t1 increments acquired with 2048 complex data points; 108 averages, and relaxation delay of 2.5 sec. Phase sensitive data was obtained by using TPPI method. Spectra were processed using TOPSPIN 3.2 version. The cross peaks of acylcarnitine (AC) resonances between β CH with α and γ CH_2_ groups are highlighted.

**Supplementary Figure S1**

**
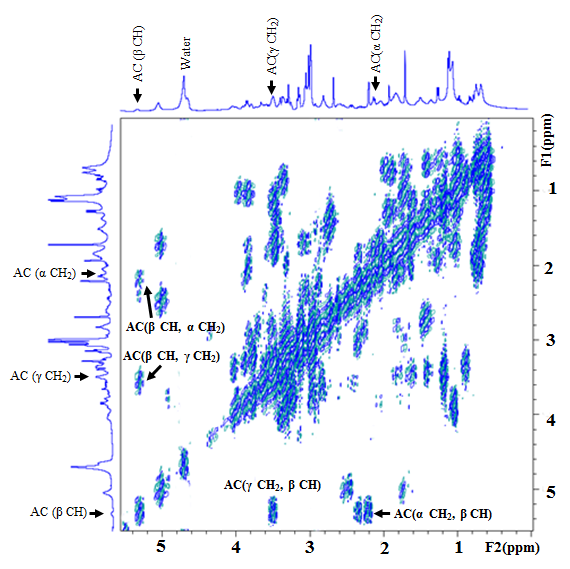
**

**Figure S1 2D HR MAS COSY spectrum of tumor tissue.**
